# Construction and application of a precise evaluation method for the quality of traditional Chinese medicine based on “target-combined quality evaluation” using safflower as an example

**DOI:** 10.3389/fphar.2025.1554895

**Published:** 2025-04-15

**Authors:** Yongfeng Zhou, Qinghua Wu, Chaoxiang Ren, Huajuan Jiang, Xulong Huang, Jiang Chen, Ping Zhang, Dingkun Zhang, Jin Pei

**Affiliations:** ^1^ Department of Pharmacy, The First Affiliated Hospital of Anhui University of Chinese Medicine, Hefei, China; ^2^ State Key Laboratory of Southwestern Chinese Medicine Resources, College of Pharmacy, Chengdu University of Traditional Chinese Medicine, Chengdu, China; ^3^ Medical Supplies Center of PLA General Hospital, Beijing, China

**Keywords:** quality evaluation, target-combined quality evaluation, safflower, biopotency, network pharmacology, metabolomics

## Abstract

**Introduction:**

The quality of traditional Chinese medicine (TCM) guarantees clinical efficacy. At present, although chemical quality evaluation methods can reflect the quality of TCMs to a certain extent, there are still many problems. This study proposes a new strategy for the quality innovation evaluation of the TCM “target combined quality evaluation” (TCQE) theory and method. Taking the “disease-target-medicine” combination as the main fusion strategy, using drug components/ components to achieve the overall intervention of disease targets, and establishing the TCQE strategy of TCM to improve the pertinence and accuracy of the quality evaluation of TCM, the clinical efficacy and safety of TCM is improved, and precision clinical medication is achieved.

**Methods:**

Taking safflower as the research object, due to its main efficacy in the treatment of primary dysmenorrhea (PD), the potential targets and mechanism of action of safflower in the treatment of PD were analyzed by a network pharmacology method and combined with a metabolomics method. The pathway and mechanism of action of safflower in the treatment of PD were analyzed from multiple dimensions. The network pharmacology and metabolomics results were analyzed, the key targets of safflower in the treatment of PD were screened. Finally, a method for the biological evaluation of safflower quality was established based on the selected targets, which could provide a reference for the precise evaluation of safflower treatment and a scientific basis for the clinical application of safflower in the prevention and treatment of PD.

**Results and discussion:**

The pharmacodynamic results showed that Safflower significantly improved Prostaglandin E2 (PGE2) and Prostaglandin F2 α (PGF2 α) levels, the PGF2 α/PGE2 ratio and the analgesic rate in PD rats. The results of network pharmacology combined with metabolomics analysis showed that Prostaglandin G/H synthase 2 (PTGS2) and arachidonic acid (AA)were important targets and pathways of Safflower in the treatment of PD. A biological evaluation method was established around PTGS2. The results of the methodological investigation showed that the method was stable and reliable. In vivo validation experiments showed that the results of this method were consistent with the pharmacodynamic results, which proved its accuracy. PTGS2 is an important target of safflower in the treatment of PD. The biological evaluation method established around PTGS2 can accurately evaluate the quality of Safflower in the treatment of PD, which also proves that the target discrimination theory proposed in this study is scientific.

## Introduction

The quality of traditional Chinese medicine (TCM) guarantees clinical efficacy, so it is crucial to accurately evaluate the quality of TCM. At present, chemical evaluation is an important method for evaluating the quality of TCMs, mainly through qualitative and quantitative analysis of individual indicator components to evaluate their quality (Zhang et al., 2022). However, although modern analytical techniques can accurately determine the content of chemical components, due to the complexity of TCM ingredients, although the content of chemical components can be accurately measured, it is still difficult to accurately determine their therapeutic effects. The famous TCM *Panax notoginseng* (PN) is a popular drug for global research. With the efforts of scientists around the world, thousands of chemical components have been identified and analyzed, making it a relatively clear TCM drug. However, the chemical components of PN have not been fully characterized, and there are still active ingredients that have not been identified. In addition, there are various clinical effects of PN, and the corresponding substance basis for different effects also has certain differences. For example, the main active ingredients in PN that have blood-activating effects are total saponins and quercetin, while the main active ingredients that have hemostatic effects are PN saponins and ginsenoside Rb1 (Wang et al., 2021; Liu et al., 2021; Zhang et al., 2019; Li et al., 2022). Therefore, how should we accurately evaluate its quality?

From the perspective of modern medicine, the core of TCM that plays a role in the treatment of diseases is chemical components, and its essence is the interaction between chemical components and disease targets. As TCM is a complex system, its components have not been fully resolved, so it is difficult to evaluate the quality of its treatments through chemical analysis. Thus, is it possible to evaluate the quality of TCM from a different perspective, starting from the disease itself, through the overall effect of TCM on disease-related targets?

Therefore, this paper proposes a quality evaluation method for TCMs based on a “target-combined quality evaluation” (TCQE), that is, to evaluate the quality of TCMs by evaluating their effect on disease-associated targets. The advantage of the TCQE is that it can reflect not only the integrity of TCM but also the mechanism by which TCM treats diseases and reflects its clinical efficacy.

Safflower has been an important gynecological medicine since ancient times, and eliminating menstrual pain is one of its important effects (Weng et al., 2021). Using safflower as a model drug, this paper established a precise method for evaluating safflower quality based on the TCQE and verified its accuracy. First, network pharmacology, metabolomics and other methods were used to analyze the main targets and pathways affected by safflower in the treatment of primary dysmenorrhea (PD), and the results were further verified by molecular biology methods. Using the selected targets as targets, a biological evaluation method was established and applied to evaluate the quality of safflower, after which the accuracy of the method was verified.

## Materials and method

### Instruments

A Thermo Vanquish (Thermo Fisher Scientific, United States) ultra-performance liquid phase system, ACQUITY UPLC^®^ HSS T3 (2.1 × 150 mm, 1.8 µm) (Waters, Milford, MA, United States) and a SpectraMax iD3 multifunctional microplate reader (MD, United States) were used.

Micropipette (Eppendorf, Germany), Ultrapure distilled water was prepared using a Millipore Milli-Q-Plus system (Millipore, Bedford, MA, United States). Screening kits for COX-2 (PTGS2) inhibitors were purchased from Beyotiome Biotechnology (Shanghai, China). The fluorescence values were measured by Fluorescence microplate reader (GEMINIXS, United States) and the SOFTmaxPRO software was the production of Molecular Devices Company in United States, Ultrasound (Nanjing, China).

### Animals

A total of 48 SPF female SD rats weighing 220 ± 20 g were purchased from Hunan SJA Laboratory Animal Co., Ltd. (Certificate No. SCXK (Xiang) 2019-0004). The experimental animals were housed in the Experimental Animal Center of Chengdu University of Traditional Chinese Medicine. The room temperature was adjusted to 24°C ± 1°C, and the humidity was 50% ± 5%. The rats were adaptively housed for 7 days before the experiment, during which they could eat and drink freely. All experiments and operations were carried out in accordance with the “Regulations on the Administration of Laboratory Animals” issued by the National Science and Technology Commission of China and approved by the Animal Experiment Ethics Committee of Chengdu University of Traditional Chinese Medicine (2021-60).

### Sample preparation

Safflower samples were collected by the research group and identified by Prof. Peijin of Chengdu University of Traditional Chinese Medicine as the dried flower of the Compositae plant *Carthamus tinctorius* L. The specific place of production for each sample is listed in Table 1.

**TABLE 1:** The information of Safflower sample.

No.	Source	No.	Source
S1	Jianyang, Sichuan province	S10	Tacheng, Xinjiang province
S2	Jianyang, Sichuan province	S11	Tacheng, Xinjiang province
S3	Jianyang, Sichuan province	S12	Tacheng, Xinjiang province
S4	Ya’an, Sichuan province	S13	Lijiang, Yunnan province
S5	Ya’an, Sichuan province	S14	Lijiang, Yunnan province
S6	Ya’an, Sichuan province	S15	Lijiang, Yunnan province
S7	Yili, Xinjiang province	S16	Lijiang, Yunnan province
S8	Yili, Xinjiang province	S17	Lijiang, Yunnan province
S9	Yili, Xinjiang province		

In each group, an appropriate amount of safflower powder was weighed; 12 times the amount of water was added; the sample was extracted by ultrasonication for 30 min/time; the sample was extracted twice; and the filtrate was combined, concentrated, and lyophilized for use.

### Network pharmacological analysis

#### Screening of effective components of safflower and prediction of potential targets

The traditional Chinese Medicine System Pharmacology analysis platform (TCMSP, http://lsp.nwu.edu.cn/tcmsp.php) was used. According to the criteria of an oral bioavailability (OB) ≥ 30% and a drug class (DL) ≥ 0.18, the active ingredients in safflower were retrieved. The effective components of safflower obtained by screening will pass the SwissTargetPrediction database (http://www.swisstargetprediction.ch/), predict potential targets of safflower, screen targets with pharmacodynamic gene matching probability scores greater than 0.1, obtain the UniProt ID of each target, remove duplicates, and establish a safflower drug target database. The candidate active ingredients were supplemented by a literature search, and while hydroxysafflor yellow A was not selected, it had many literature reports and was included in the Pharmacopoeia Accusation Index.

#### Acquisition of disease targets

The GeneCards database was used to search for genes related to PD disease with the keyword “primary dysmenorrhea”. Genes with a score greater than the median were screened and converted to the corresponding disease targets through the UniProt database.

#### “Drug component target protein disease” network construction

The network and component action target information were imported into Cytoscape 3.5.1 software; the species was defined as “*Homo sapiens*”; the “drug component target disease” visual network diagram of safflower was constructed, and the attributes of each node were marked with shape and color, where the red triangle was the component of safflower, the sky blue circle was the drug target (not PD target), the yellow circle was the PD disease target (not drug target), and the yellow square was the common target of drug and PD.

### Network target analysis of the effect of safflower on PD

The network analyzer module of the Cytoscape software was used to analyze the topology of safflower “drug component common target disease” network. According to the degree, betweenness centrality and closeness centrality of network nodes, PD-related disease targets were screened to obtain the network targets of safflower in PD.

### KEGG enrichment analysis

Using the DAVID bioinformatics resource database (https://david.ncifcrf.gov/), the network targets were annotated with KEGG (Kyoto Encyclopedia of Genes and Genomes) pathways, enriched for KEGG pathways, and visualized by drawing bubble plots using bioinformatics (http://bioinformatics.com.cn/).

### Metabonomics analysis

#### Model establishment

Forty-eight female SD rats were randomly divided into six groups (n = 8): the control group, model group, positive drug group, and high-, medium-, and low-dose safflower groups (0.4 g/kg, 0.2 g/kg, 0.1 g/kg). Except for the blank control group, which was subcutaneously injected with normal saline, the PD models were established in the other groups: estradiol benzoate (0.35 mg/kg) was subcutaneously injected daily for seven consecutive days. The experimental group were gavaged safflower, and the control group and model group were given the same amount of normal saline during this period. The Yang medicine group was given celecoxib (40 mg/kg). Oxytocin (0.01 mL/g) was injected intraperitoneally 30 min after the last administration. The participants fasted for 12 h before the experiment and could not drink freely (Wang et al., 2022; Cheng et al., 2018).

### Sample pretreatment

The experimental sample was shaken at 4°C, vortexed for 1 min after thawing, and mixed evenly. An appropriate amount of sample was accurately transferred to a 2 mL centrifuge tube, 400 µL of methanol solution (−20°C) was added, and the sample was vortexed for 1 min. The sample was centrifuged at 12,000 rpm at 4°C for 10 min, and the entire supernatant was transferred to a new 2 mL centrifuge tube, concentrated and dried. Then, 150 µL of 2-chloro-l-phenylalanine (4 ppm) solution prepared with 80% methanol water (stored at 4°C) was added to redissolve the sample, and the supernatant was removed after 0.22 μM membrane filtration. The filtered solution was added to the detection bottle for LC‒MS detection.

### LC/MS analysis conditions

Chromatographic conditions: a flow rate of 0.25 mL/min, a column temperature of 40°C, and an injection volume of 2 μL. In positive ion mode, the mobile phase was 0.1% formic acid acetonitrile (B2) and 0.1% formic acid water (A2), and the gradient elution procedure was as follows: 0–1 min, 2% B2; 1–9 min, 2%–50% B2; 9–12 min, 50%–98% B2; 12–13.5 min, 98% B2; 13.5–14 min, 98%–2% B2; and 14–20 min, 2% B2. The mobile phase was acetonitrile (B3) and 5 mM ammonium formate water (A3), and the gradient elution procedure was as follows: 0–1 min, 2% B3; 1–9 min, 2%–50% B3; 9–12 min, 50%–98% B3; 12–13.5 min, 98% B3; 13.5–14 min, 98%–2% B3; and 14–17 min, 2% B3.

The mass spectrometry conditions used were as follows: Thermo Orbitrap Explorer 120 mass spectrometer detector (Thermo Fisher Scientific, United States), electrospray ionization (ESI) source, and positive and negative ion mode for data collection. The positive ion spray voltage was 3.50 kV; the negative ion spray voltage was −2.50 kV; the sheath gas was 30 ARB, and the auxiliary gas was 10 ARB. The capillary temperature was 325°C; the first-order full scan was performed with a resolution of 60,000; the first-order ion scanning range was 100∼1,000 m/z; HCD was used for second-order cleavage; the collision energy was 30%; the second-order resolution was 15,000; the first four ions of the signal were collected for fragmentation, and dynamic exclusion was used to remove unnecessary ms/ms information.

### Data processing and analysis

The original mass spectrometry offline files were converted to mzXML file format by the msConvert tool in the ProteoWizard software package (v3.0.8789). The Rxcms software package was used for peak detection, peak filtration and peak alignment to obtain a quantitative list of substances. The parameter settings were bw = 2, ppm = 15, peakwidth = c (5,30), mzwid = 0.015, mzdiff = 0.01, and method = “centwave”. The public databases HMDB, MassBank, LIPID MAPS, mzCloud, KEGG, etc., were used for the identification of substances, and the parameters were set as ppm < 30 ppm. The Loess signal correction method based on QC samples realizes data correction and eliminates system errors. Substances with an RSD > 30% in the QC samples were filtered out for data quality control.

Principal component analysis (PCA) and orthogonal partial least squares discriminant analysis (OPLS-DA) dimension reduction analysis were performed on the sample data using SIMCA software. A score map, load map and volcano map were drawn to analyze the differences in metabolite composition among the samples. The permutation test method was used to test the fit of the model.

Differentially abundant metabolites with P < 0.05 and VIP >1 were screened as screening criteria. Pathway analysis was performed using MetaboAnalyst (https://www.metaboanalyst.ca/). The data analysis platform performs functional pathway enrichment and topology analysis for screening differential metabolic molecules.

### Biopotency assay

Celecoxib was used as the standard reference material and safflower reference material (S1) was diluted with four kinds of concentrations at the ratio of 1:0.5. The inhibition rate of PTGS2 was measured by three respectively. The original biopotency of reference material of safflower was defined as 1,000 U/μg. Other samples of different batches are test group. According to the principle of simple probability unit method, the biopotency and confidence limit rate (FL) of inhibiting PTGS2 activity are calculated.

## Results

### Network pharmacology screening of the targets and pathways affected by safflower in the treatment of PD

A total of 189 chemical components of safflower were obtained from the TCMSP database. According to an oral bioavailability (OB) ≥ 30% and a drug class (DL) ≥ 0.18, 22 active components meeting the requirements were ultimately screened out. In addition, 23 components of the quality control index hydroxysafflor yellow A in the Pharmacopoeia have been reported in the literature. The SwissTargetPrediction database was used for component target prediction, and a total of 222 potential targets of 22 components were obtained.

**FIGURE 1:**
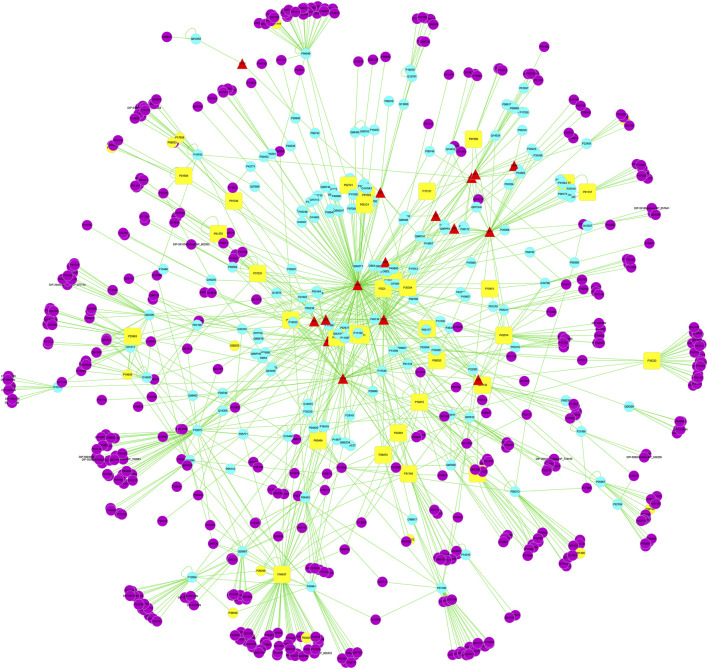
The compound-target-disease interaction network of Safflower for treating PD. The yellow square represents the joint target of drugs and diseases, which is also the most important direct target protein in treating inflammation by Safflower. Yellow dots represent PD targets. The red triangle represents the predicted possible active ingredients in Safflower. The blue dot represents the target of Safflower. The purple dots represent the interaction between the target proteins and the active components of Safflower.

A total of 230 PD-related targets were obtained from the GeneCards database, and the correlation score ranged from 0.676 to 37.068, with a median of 6.378. Those above the median were selected as the main PD targets, and a total of 116 PD disease targets were obtained. Cytoscape 3.5.1 software was used to construct the active ingredient target disease network of safflower for the treatment of PD (Figure 1). In the active ingredient target disease network of safflower for the treatment of PD, the median node degree, intermediate centrality, and near centrality were 36.5, 0.0014, and 0.3, respectively. For core nodes with node degree values greater than twice the median, the key targets with intermediate centrality and near centrality not lower than the median were screened. The results showed that 33 targets, including cellular tumor antigen p53, Caspase-3, apoptosis regulator Bcl-2, catenin beta-1, NF-kappa-B inhibitor alpha, RAC alpha serine/threonine protein kinase, NF-kappa-B inhibitor alpha, vascular endothelial growth factor A and PTGS2, may be the key targets of safflower in the treatment of PD (Table 2).

**TABLE 2:** Potential target protein information of Safflower in the treatment of PD (top 20).

No.	Uniprot ID	Protein names	Closeness centrality	Degree	Betweenness centrality
1	P04637	Cellular tumor antigen p53	0.3836	63	0.1359
2	P42574	Caspase-3	0.3745	24	0.0439
3	P10415	Apoptosis regulator Bcl-2	0.3796	23	0.0518
4	P35222	Catenin beta-1	0.2855	23	0.0569
5	P25963	NF-kappa-B inhibitor alpha	0.3555	19	0.0297
6	P31749	RAC-alpha serine/threonine-protein kinase	0.3664	18	0.0352
7	P15692	Vascular endothelial growth factor A	0.3534	16	0.0234
8	P35354	Prostaglandin G/H synthase 2	0.3760	14	0.0232
9	P37231	Peroxisome proliferator activated receptor gamma	0.3539	12	0.0147
10	P01137	Transforming growth factor beta-1	0.3350	10	0.0189
11	P01375	Tumor necrosis factor	0.3475	10	0.0159
12	Q03135	Caveolin-1	0.3576	10	0.0181
13	P23219	Prostaglandin G/H synthase 1	0.3650	9	0.0097
14	P07550	Beta-2 adrenergic receptor	0.3381	6	0.0117
15	P14780	Matrix metalloproteinase-9	0.3449	6	0.0042
16	P01344	Insulin-like growth factor II	0.3294	5	0.0095
17	P29474	Nitric-oxide synthase, endothelial	0.3303	5	0.0071
18	P08253	72 kDa type IV collagenase	0.3449	4	0.0045
19	P22301	Interleukin-10	0.3393	4	0.0065
20	P01584	Interleukin-1 beta	0.3236	3	0.0060

### Screening of the safflower treatment pathway for PD based on metabonomics

#### Pharmacodynamics of safflower in improving PD in rats

**FIGURE 2:**
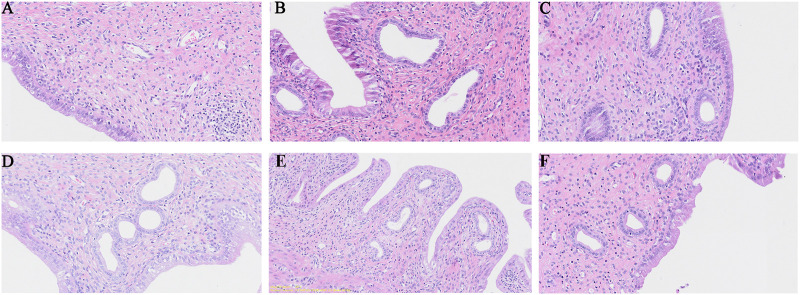
H&E staining of rat uterine tissues (×10) from normal control group **(A)**, model group **(B)**, Celecoxib **(C)**, Safflower-Low **(D)**, Safflower-Middle **(E)** and Safflower-High group **(F)**.

Pathology revealed that the epithelial structure of the uterine mucosa in the blank group was complete, that the epithelial cells were normal and closely arranged, that the stroma of the lamina propria was dense, that the number of uterine glands was abundant and that there was no inflammatory reaction (Figure 2A). In the model group, a small amount of cytoplasmic vacuolation, apoptosis and nucleolysis of mucosal epithelial cells, obvious expansion of uterine glands in the lamina propria, an obvious reduction in the number, and diffuse inflammatory cell infiltration were observed (Figure 2B). In the celecoxib group, the morphology and structure of the uterine epithelial cells returned to normal, but obvious uterine gland expansion was still visible locally (Figure 2C). After safflower treatment, cellular inflammation significantly improved, the cytoplasmic vacuolation of epithelial cells decreased, and the expansion of uterine glands in the lamina propria significantly improved. The effect in the high-dose safflower group was greater than that in the low-dose safflower group (Figures 2D–F).

**FIGURE 3:**
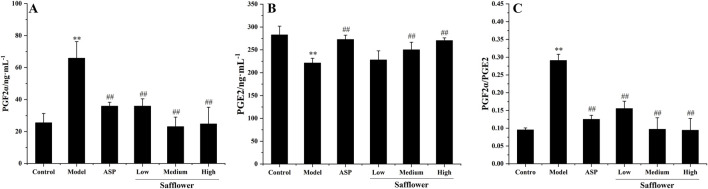
Effect of Safflower on biochemical indices of PGF2α and PGE2 in PD rats. **(A)** The results of Safflower on serum biochemistry of PGF2α in PD rats, **(B)** The results of Safflower on serum biochemistry of PGE2 in PD rats, **(C)** The results of Safflower on serum biochemistry of PGF2α/PGE2 in PD rats.

Compared with that in the control group, the PGF2α level in the model group was significantly greater than that in the blank group. The content of PGE2 increased significantly, and the content of PGE2 decreased significantly (P < 0.01), indicating that the model was successfully established. Compared with those in the model group, the PGF2α levels in each group after administration and PGE2 levels were significantly changed (p < 0.01). Compared with those in the control group, the level of PGF2α increased, and the level of PGE2 decreased significantly (p < 0.01), indicating that the model was successfully established. Safflower at different concentrations significantly reduced PGF2α levels, increased PGE2 levels, and reduced the level of PGF2 α/PGE2 (p < 0.01), suggesting that safflower has a significant effect on the treatment of PD and that the effect of a high dose is equivalent to that of positive drugs (Figures 3A–C).

#### Effects on plasma metabolites

**FIGURE 4:**
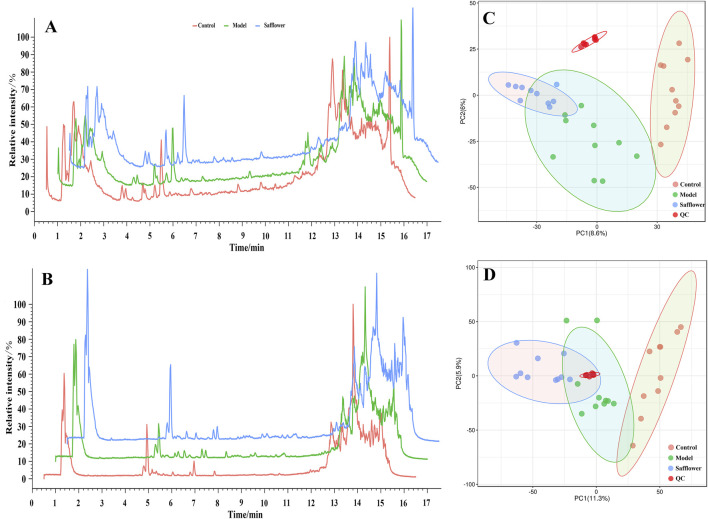
Total ion flow diagram and PCA analysis. **(A)** Total ion flow diagram of different groups in positive ion mode; **(B)** Total ion flow diagram of different groups in negative ion mode; **(C)** PCA analysis of different groups in positive ion mode; **(D)** PCA analysis of different groups in negative ion mode.

The PCA results showed that, under the positive and negative ion modes, the normal control group, the model group and the administration group all had good discrimination, indicating that there were large differences in the metabolites of these three groups (Figure 4).

**FIGURE 5:**
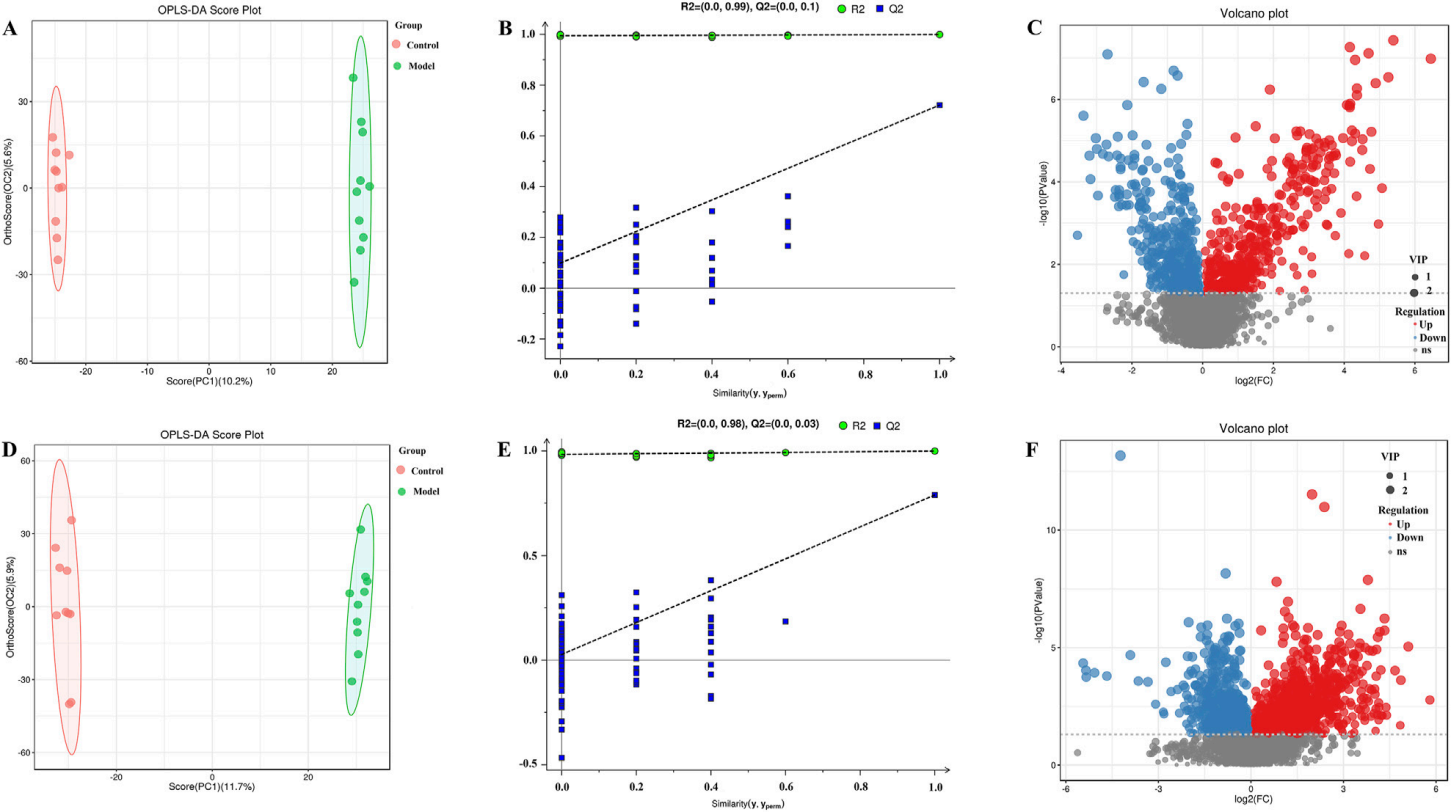
OPLS-DA score plot, validation plot and volcano plot of plasma samples in control group and model group. **(A–C)** are in positive ion mode, **(D–F)** in negative ion mode.

**FIGURE 6:**
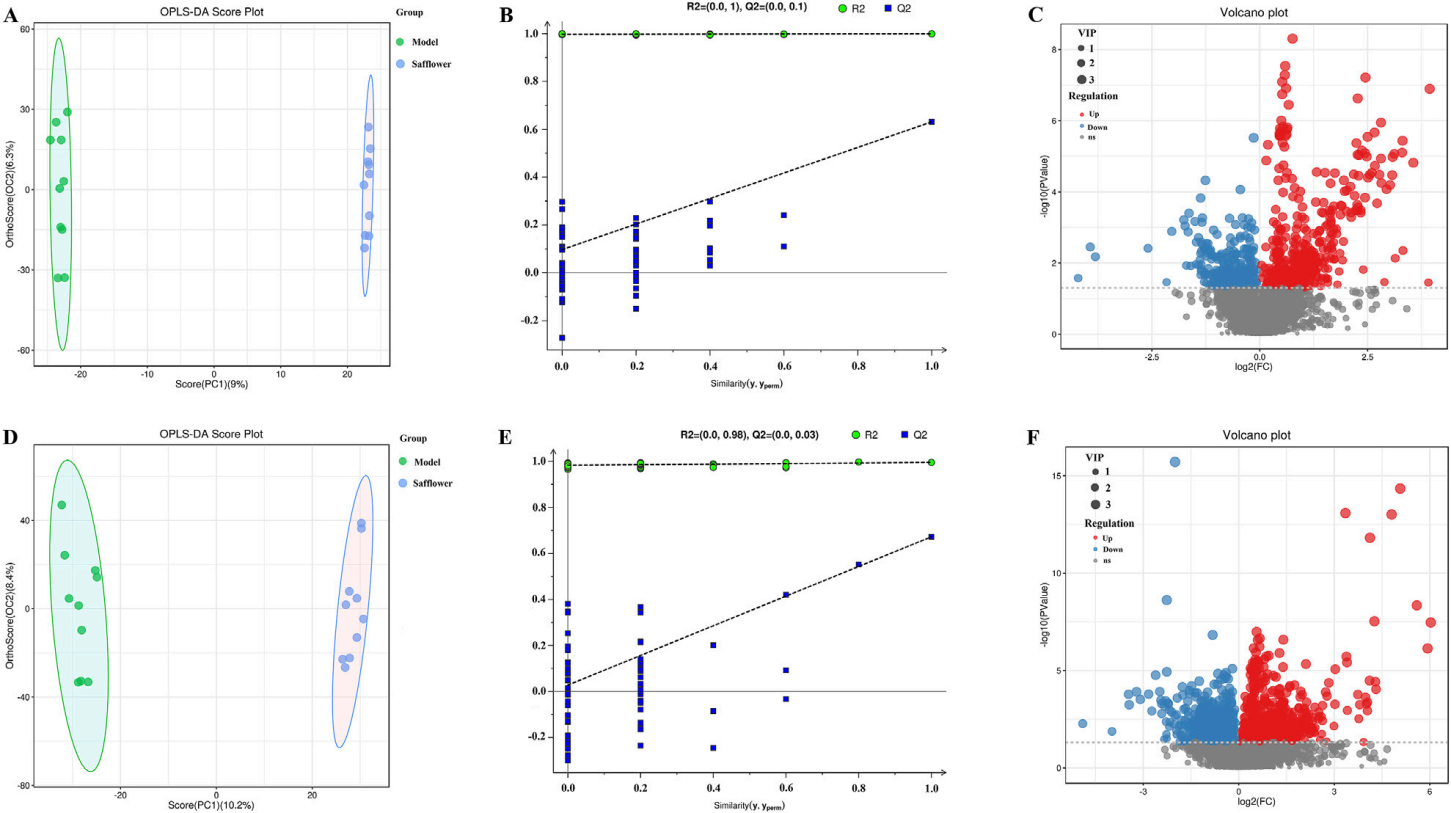
OPLS-DA score plot, validation plot and volcano plot of plasma samples in model group and Safflower group. **(A–C)** are in positive ion mode, **(D–F)** in negative ion mode.

OPLS-DA was carried out for the blank, model, model and treatment groups, and the results are shown in Figures 5A, D, 6A, D. The results showed that the blank group and the model group as well as the model group and the administration group could be significantly distinguished. The permutation test was used to estimate the robustness and predictive ability of our model, the values of Q2 (Qmax2 < Q2) intercept indicated that the OPLS-DA model had not been overfitted, and these models were considered reliable and predictable. Therefore, further differential metabolite analyses were performed based on these separation results (Figures 5B, E, 6B, E).

Volcano plots of differentially abundant metabolites are shown in Figures 5C, F, 6C, F. Each point in the volcano map represents a metabolite, and the map contains all substances measured in this experiment. The abscissa represents the fold change of the group compared with each substance (taking the logarithm of base 2); the ordinate represents the p value of the t-test (taking the negative number of base logarithm of base 10), and the scatter size represents the VIP value. The larger the scatter is, the greater the VIP value. Significantly upregulated metabolites are indicated in red; significantly downregulated metabolites are indicated in blue, and nonsignificantly different metabolites are indicated in gray.

**FIGURE 7:**
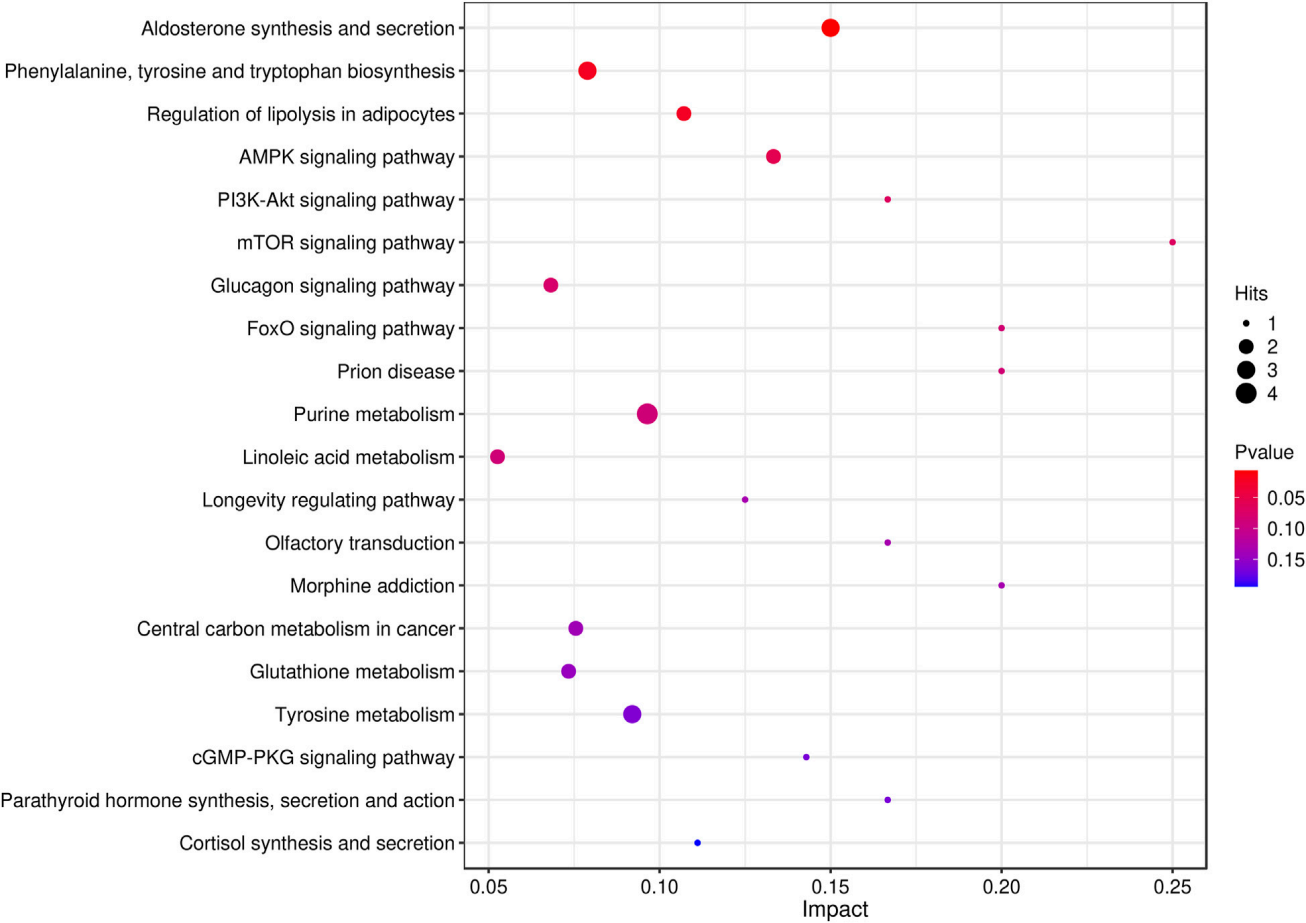
The result of metabolic pathway analysis (top 20).

A total of 52 metabolic pathways, including purine metabolism and arachidonic acid metabolism, were obtained by CO enrichment analysis of differentially abundant metabolite entry pathways. The results showed that the protective effect of safflower on a rat primary dysmenorrhea model was achieved through different metabolic pathways (Figure 7).

#### Target screening for safflower in the treatment of PD

**FIGURE 8:**
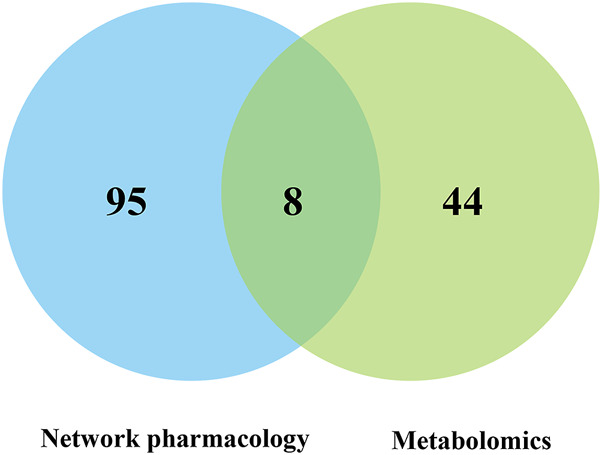
Common pathway analysis of metabolomics and network pharmacology.

The integrated analysis of pathways enriched by network pharmacology and metabolomics showed that there were eight metabolic pathways in network pharmacology and metabolomics, which were the PI3K-Akt signaling pathway, longevity regulating pathway, regulation of lipolysis in adipocytes, pathways of neurogenesis in multiple diseases, FOXO signaling pathway, prion disease, arachidonic acid metabolism, and central carbon metabolism in cancer (Figure 8).

The top 10 targets screened by the Institute of Network Pharmacology were imported into KEGG to query the associated pathways. The results showed that the PTGS2 target was associated with arachidonic acid metabolism and was the target of this pathway. The RAC alpha serine/threonine protein kinase target is related to the FOXO signaling pathway and is the target of this pathway.

A literature survey revealed that the arachidonic acid pathway is closely related to the occurrence of inflammation and is an important pathway in PD, while the FOXO signaling pathway mainly plays an important role in tumorigenesis, lifespan regulation, metabolic regulation, etc. Therefore, this study focused on the PTGS2 target in the arachidonic acid pathway.

### Construction of a method for the biological evaluation of safflower quality based on target proteins

Taking the PTGS2 target as the core, a method for evaluating the clinical efficacy of safflower quality was established by using the PTGS2 Enzyme Activity Kit detection method. The methodological investigation revealed that the precision RSD value was between 4.56% (n = 5), that the repeatability RSD value was 4.93% (n = 5) and that the stability RSD value was 3.79% (n = 5), indicating that the method was stable and reliable.

**FIGURE 9:**
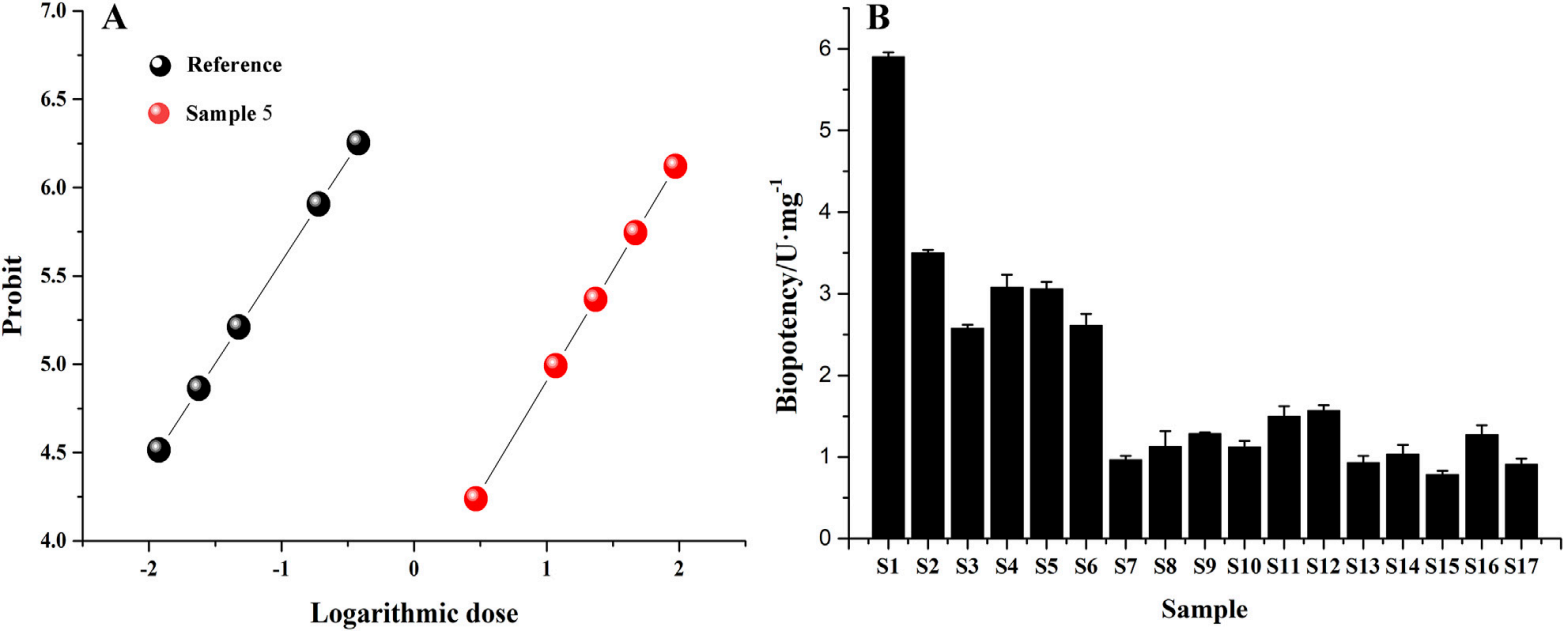
Analysis of biopotency results of Safflower inhibiting PTGS2 activity. **(A)** The relationship between logarithmic doses and probit; **(B)** biological potency of safflower samples from different batches.

The concentration and inhibition rate of 17 batches of safflower were input into the Chinese Medicine Potency Calculation Software; the positive control drug celecoxib was used as a reference, and its potency was calculated as 100 U/mg. The results showed that the biopotency of different batches of safflower greatly differed. Among them, the biopotency of sample S1 was the greatest, while the biopotency of sample S15 was 6.5 times lower (Figure 9).

### Accuracy verification

**FIGURE 10:**
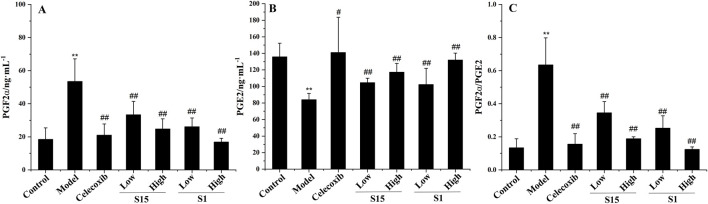
Therapeutic effect of two groups of Safflower samples on PD. **Compared with the control group, P < 0.01, * compared with the control group, P < 0.05, ## Compared with the model group, P < 0.01, # compared with the model group, P < 0.05. **(A)** The results of Safflower on serum biochemistry of PGF2α in PD rats, **(B)** The results of Safflower on serum biochemistry of PGE2 in PD rats, **(C)** The results of Safflower on serum biochemistry of PGF2α/PGE2 in PD rats.

To further verify the accuracy of safflower biopotency determination, a comparative study of two groups (S1 and S15) of safflower samples based on the PD model was carried out. The results showed that both groups of safflower samples could improve the relevant biochemical indicators of the PD model, and the effect of safflower samples in the higher biopotency group was due to the safflower samples in the lower biopotency group, which preliminarily proved that the safflower quality evaluation based on this method was accurate (Figure 10).

## Discussion

The quality of TCM is its guarantee of clinical efficacy. As the era of precision medicine has developed, precise evaluation of the quality of TCM has become an important goal of the quality evaluation and control of modern TCM. Unlike chemical drugs, TCM has “multicomponent, multitarget and multiefficacy” characteristics. Therefore, determining how to achieve accurate evaluation is crucial. The author believes that the accurate evaluation of the quality of traditional Chinese medicine includes two aspects: the “precision” of the evaluation method refers to the good precision and minimal error of the method, and the “accurate” evaluation method refers to good accuracy, which can accurately reflect a treatment’s clinical efficacy. In short, accurate evaluation of the quality of traditional Chinese medicine should not only accurately correlate with clinical efficacy but also have good precision (Zhou et al., 2023).

Compared with traditional chemical analysis, biological evaluation has the advantage of correlation efficacy, but it also has some shortcomings. Biological evaluation methods mostly rely on animals, cells and *ex vivo* tissues and organs, which have the disadvantages of high experimental detection cost and complex operation. In addition, the lack of precision also seriously restricts its application in actual production and inspection processes. Based on biological evaluation, this study transformed animal or cell test methods into kit detection methods, which greatly improved the simplicity and precision of biological evaluation methods (Li et al., 2017; Tilton et al., 2010; Song et al., 2023).

Recent research has shown that the abnormal increase in uterine prostaglandin (PG) levels is one of the important mechanisms of PD. Abnormal secretion of PGs can lead to uterine contraction, ischemia, inflammation and pain (Paloma et al., 2019). Studies have shown that the abnormal release of PGE2 by PGF2α leads to smooth muscle spasm and contraction, endometrial detachment, and bleeding and pain, while PGE2 can inhibit uterine smooth muscle contraction (Ma et al., 2021; Xie et al., 2022). PTGS2 is an important rate-limiting enzyme involved in the synthesis of PGs. The elevated expression of PTGS2 before menstruation can catalyze the conversion of arachidonic acid into active PGs, resulting in spastic pain. Current clinical drugs for PD, such as ibuprofen, reduce the synthesis of PGs and relieve pain by specifically inhibiting PTGS2 (Tang et al., 2020).

This study also has several shortcomings. In this study, network pharmacology combined with metabolomics was used to screen out some targets, but only PTGS2 was verified. In addition, the established kit-based detection method was developed on the basis of the existing kit, and no new kit has been developed.

## Conclusion

Accurate evaluation of the quality of TCM should aim to accurately reflect clinical efficacy, selecting appropriate indicators, establishing appropriate methods, and comprehensively and accurately reflecting its efficacy. This study takes the clinical efficacy of TCM as the goal, follows the basic theoretical framework of TCM, uses modern analytical techniques and methods to analyze the mechanism of disease treatment, integrates the biological evaluation of TCM quality, constructs a quality evaluation method for TCM based on “target identification and quality evaluation, breaks the original research habit of chemical evaluation of TCM quality, enriches and develops a quality evaluation system for TCM, provides references for the accurate evaluation of TCM quality, and provides new ideas and methods for research on TCM quality evaluation.

## Data Availability

The raw data supporting the conclusions of this article will be made available by the authors, without undue reservation.
